# Supplementary material for: Long-range spatial extension of exciton states in van der Waals heterostructure
Source: Nat Commun. 2026 Mar 5;17:3503. doi: 10.1038/s41467-026-70218-4 (PMC13083923; doi:10.1038/s41467-026-70218-4)
Supplement: Supplementary file 1 — Supporting Information for Long-range Spatial Extension of Exciton States in Van der Waals Heterostructure [file 41467_2026_70218_MOESM1_ESM.pdf]

Supporting Information for
Long-range spatial extension of exciton states in van der Waals heterostructure

Zhiwen Zhou,[1] E. A. Szwed,[1] W. J. Brunner,[1] H. Henstridge,[1] L. H. Fowler-Gerace,[1] and L. V. Butov[1]
[1]*Department of Physics, University of California San Diego, La Jolla, CA 92093, USA*

Heterostructure

The $MoSe_2/WSe_2$ heterostructure (Fig. S1a) was assembled using the dry-transfer peel technique [1]. The manufacturing details are described in Ref. [2] where the same heterostructure was used for studies of IX transport. The thickness of the bottom hBN layer is ∼ 40 nm, the thickness of the top hBN layer is ∼ 30 nm. The $MoSe_2$ monolayer is on top of the WSe_2 monolayer. The long WSe_2 and $MoSe_2$ edges (Fig. S1b) enable a rotational alignment between the WSe_2 and $MoSe_2$ monolayers. The twist angle between the monolayers $\delta\theta = 1.1°$ corresponding to the moiré superlattice period $b = 17$ nm agrees with the angle between $MoSe_2$ and WSe_2 edges in the heterostructure (Fig. S1b).

Figure S1b shows the layer pattern of the heterostructure. The hBN layers cover the entire areas of $MoSe_2$ and WSe_2 layers. There was a narrow multilayer graphene electrode on the top of the heterostructure around $x = 2$ μm for $y = 0$ in Fig. S1b, this electrode was detached.

So far, the long-range extension of the narrow PL lines and the corresponding exciton states was realized in one sample in this work. Other studies of GaAs and TMD heterostructures show short extension of the narrow lines, below the spatial resolution of the optical measurements, as outlined in the main text. A shorter extension of exciton states likely originates from stronger disorder. This work demonstrates the existence of the long-range extension of exciton states in TMD heterostructures. Studying this phenomenon in other samples and by other experimental techniques is the subject for future work. As outlined in the introduction in the main text, the long-range extension of the narrow PL lines is observed in the heterostructure presenting the long-range IX transport [2], the long-range IX mediated spin transport [3], and the high IX diffusivity and mean free path [4]. So far, this efficient exciton transport [2–4] was realized in one sample. Studying this efficient exciton transport in other samples and, in particular, verifying its relation to the long-range extension of the narrow PL lines is also the subject for future work.

We checked that the long-range extension of the narrow PL lines is reproducible in multiple repeated measurements. As to the energies of narrow lines, they are reproducible when the measurements are repeated within a short time (days), however, they change over longer time (months). The mechanism of this spectral diffusion is yet to be understood. The long-range extension of narrow lines survives over longer time: Narrow lines with longer extensions along x are seen as vertical modulated lines in the x−Energy maps both in Fig. 2 in the main text and in Fig. S3, with the latter showing the data measured more than a year later, in a different cryostat, and using a different laser with a different excitation spot as outlined in Section Density dependence.

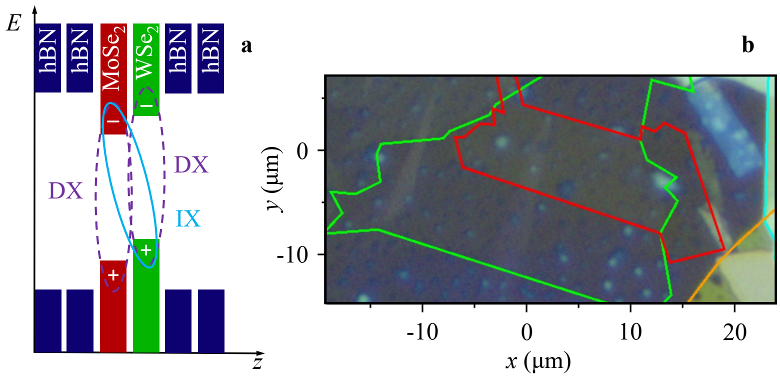

FIG. S1: (a) Energy-band diagram of the $MoSe_2/WSe_2$ heterostructure. The ovals indicate spatially direct excitons (DXs) and spatially indirect exciton (IX) composed of an electron (−) and a hole (+). (b) A microscope image showing the heterostructure layers. The green, red, cyan, and orange lines indicate the boundaries of WSe_2 and $MoSe_2$ monolayers and bottom and top hBN layers, respectively.

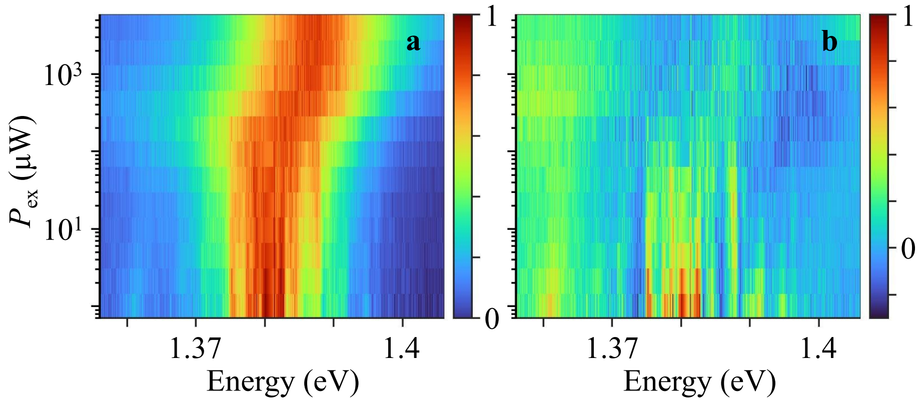

FIG. S2: (a,b) The excitation power P_{ex} dependence of IX spectra. The spectra intensities at different P_{ex} are normalized. At low P_{ex}, the narrow lines are observed on a background of a broad line. This broad-line background is subtracted from the spectra in (b). The laser excitation spot is focused to a spot ~ 2 μm in diameter. T = 3.5 K.

Optical measurements

The continuous-wave PL experiments are outlined in Section Methods in the main text.

Time resolved measurements were performed using a semiconductor laser with the excitation energy 1.689 eV and step-like rectangular pulse shape with 80 ns pulse width, 0.3 ns pulse fall time (the pulses fall e times within 0.3 ns), and 300 ns pulse period. The signal was measured by an avalanche photodiode, which was moved in the plane of the optical image to measure the PL lifetime vs. x. The temporal and spatial resolution was ~ 0.3 ns and ~ 1 μm.

The g factor measurements were performed using circularly polarized excitation and co- and cross-polarized PL signal in magnetic fields up to 8 T oriented perpendicular to the heterostructure plane. The $x-$Energy and $x-y$ images present co-polarized PL. The sample was mounted on an Attocube xyz piezo translation stage allowing adjusting the sample position relative to a focusing lens inside the cryostat.

Density dependence

Figure S2 shows the excitation power P_{ex} dependence of IX spectra, similar to the excitation power dependence in Fig. 1a,b in the main text. At low P_{ex}, the narrow lines are observed on a background of a broad line. This broad-line background is subtracted from the spectra in Fig. S2b.

The broad background in this work is approximated by the Gaussians drawn through the origin of the narrow lines as shown in Fig. 1a in the main text. In this approach, the spectrum is fitted by a broad Gaussian presenting the broad background (green Gaussian in Fig. 1a) and narrow Gaussians for each narrow line. In an alternative approach, we drew the broad Gaussians as the fits for the spectra and analyzed the deviations of the spectra from those Gaussians. The differences between the maxima and neighbor minima in the deviations presented the amplitudes of the narrow lines. This alternative approach gave similar results to the approach presented in Fig. 1a.

Figure 1c in the main text shows the relative intensity of the narrow lines presented by the ratio of the sum of spectrally integrated intensities of the narrow lines to the spectrally integrated intensity of the broad line in the PL spectrum. Figure S3a shows the sum of spectrally integrated intensities of the narrow lines and shows the spectrally integrated intensity of the broad line in the PL spectrum. The intensities of narrow lines grow slower with power than the intensity of the broad line (Fig. S3a), consistent with the data in Fig. 1c in the main text. Figures S3b-d show $x-$Energy maps for higher excitation powers. With increasing density, the energies of the narrow lines stay fixed (Fig. 3b-d), consistent with the lack of change of energies of narrow lines with density discussed in the main text.

Temperature dependence

$x-$Energy maps measured at different temperatures are shown in Fig. S4. These measurements show that the long-range extension of the narrow lines is also observed at higher temperatures, the energies of the narrow lines practically

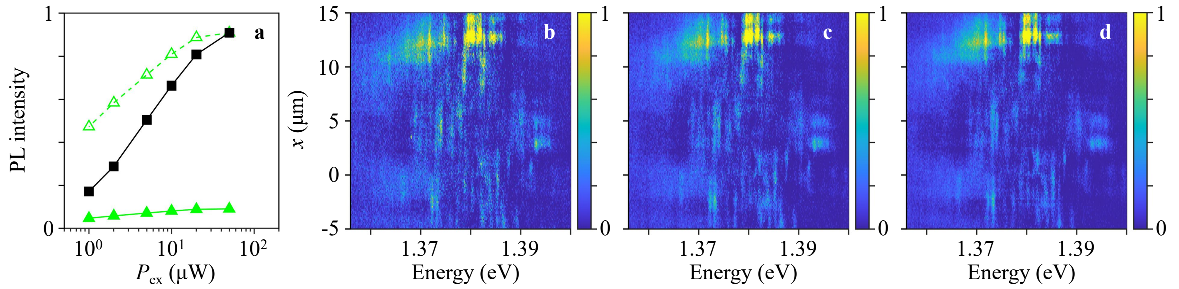

FIG. S3: (a) Figure 1c in the main text shows the relative intensity of the narrow lines presented by the ratio of the sum of spectrally integrated intensities of the narrow lines to the spectrally integrated intensity of the broad line in the PL spectrum. Figure S3a separately shows the sum of spectrally integrated intensities of the narrow lines (green triangles, the intensities ×10 are shown by open triangles) and the spectrally integrated intensity of the broad line (black squares). (b-d) $x-$Energy maps for higher excitation powers. The broad background is subtracted. The excitation spot is defocused over a spot $\sim 35 \times 45$ μm covering the heterostructure area for excitation of the entire sample. The excitation power of this defocused excitation is P_{ex} = 200 μW (b), 500 μW (c), and 1000 μW (d). T = 4.2 K.

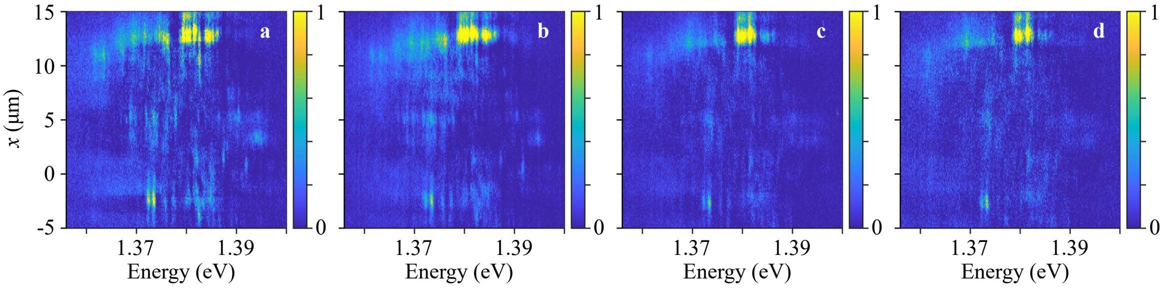

FIG. S4: $x-$Energy maps for higher temperatures. The broad background is subtracted. The excitation spot is defocused over a spot $\sim 35 \times 45$ μm covering the heterostructure area for excitation of the entire sample. The excitation power of this defocused excitation P_{ex} = 200 μW. The temperature is T = 1.7 K (a), 4.2 K (b), 8 K (c), and 10 K (d). The narrow lines quickly vanish with increasing temperature above 10 K.

do not change with temperature, and the intensities of the narrow lines reduce with increasing temperature, similarly at different locations.

Lifetime

We measured the PL lifetime using excitation by a pulsed semiconductor laser and detecting the signal by APD. In these measurements, the signal is insufficient to measure the position-dependent lifetime for a single selected narrow line. Therefore, we measured the position-dependent lifetime for spectrally integrated PL (Fig. S5). The spectral integration combines in the measurements all narrow lines in the range $E < 1.46$ eV of the PL spectrum. Lifetimes of different narrow lines may be different. The $x-$Energy maps in this work show the emergence of different narrow lines at different positions that may contribute to variations of the spectrally integrated lifetime along x. The measured lifetime varies roughly within the accuracy of the measurements along the heterostructure (Fig. S5). The measurements of the position-dependent lifetime for a single selected narrow line is the subject for future work.

g-factor

The g factor of the exciton states corresponding to the narrow PL lines is measured using circularly polarized laser excitation and co-polarized and cross-polarized PL of the narrow lines. The co-polarized PL of the narrow lines is $\sim$ 4 times stronger than the cross-polarized. Figures S6a and S6b show the energies of co-polarized E_{co} and cross-polarized E_{cross} PL of the narrow lines vs. magnetic field. Their difference allow estimating the exciton g factor:

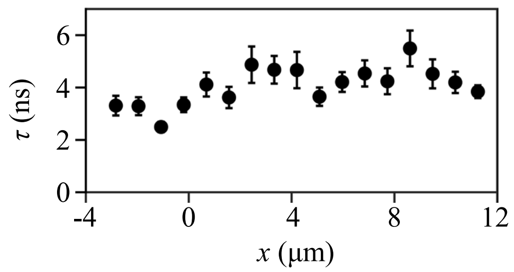

FIG. S5: Lifetimes vs. x. The PL signal is spectrally integrated in the range $E < 1.46$ eV. The lifetimes are obtained by least-squares exponential fit to the PL decay kinetics within 2 ns after the end of the laser excitation pulse. The laser excitation is focused to a spot ~ 2 μm in diameter. $P_{ex} = 50$ μW. $T = 4.2$ K.

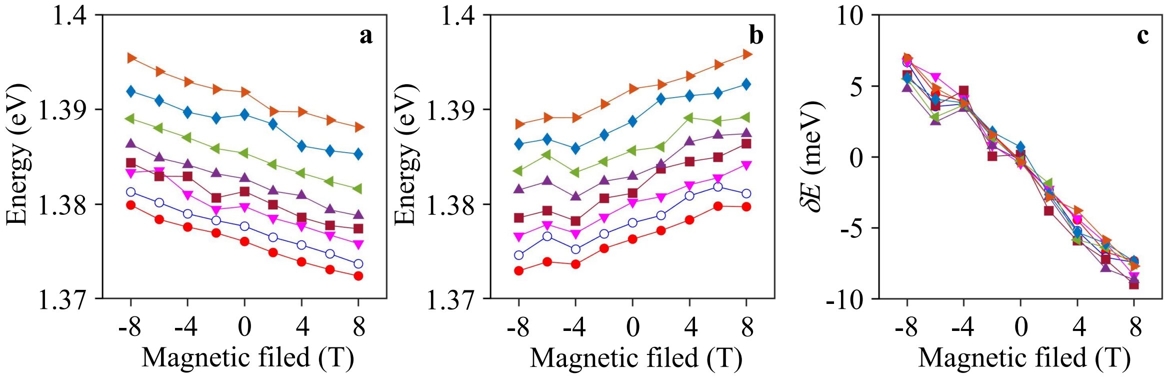

FIG. S6: g factor of narrow-line exciton states. (a,b) Energies of co-polarized E_{co} (a) and cross-polarized E_{cross} (b) emission of the narrow lines vs. magnetic field. The laser excitation is circularly polarized. (c) The energy difference $\delta E = E_{co} - E_{cross}$ for the narrow lines vs. magnetic field B. The same symbol and color is used for a certain narrow line in (a), (b), and (c).

$\delta E = E_{co} - E_{cross} = g\mu_B B$, where μ_B is the Bohr magneton. Figure S6c shows that for all narrow lines, the measured excitonic g factor is $g \sim -15.5 \pm 0.7$. The measured $g \sim -15.5 \pm 0.7$ corresponds to H_h^h site in the moiré potential of the $MoSe_2/WSe_2$ heterostructure with, in turn, H stacking [5, 6].

x−Energy maps

Figure S7 shows x−Energy maps of the exciton PL. This figure is similar to Fig. 2 in the main text, however, it shows x−Energy maps for more y locations in the heterostructure. In these maps, the narrow lines are revealed by the spectrally narrow enhancements of the PL intensity. In Fig. 2 in the main text and in Fig. S7, the broad background (given by Gaussians in Fig. 1a in the main text) is subtracted. x−Energy maps without background subtraction are shown in Fig. S8.

Lineshape

Figures S9a and S9b show the x−Energy map and the corresponding spectra of narrow lines at various x positions. The lineshapes of the narrow lines are fit by the narrow Gaussians in Fig. S9c. Figure S9d shows the detailed spectra at various x positions for the narrow line at 1.3768 eV, which extends over long distances. The detailed lineshapes show nearly identical energy and linewidth of the narrow line at various x positions. The measured lineshapes may be useful for future theoretical work aiming to model PL line shapes, spectral diffusion, and exciton diffusion characteristics in this system.

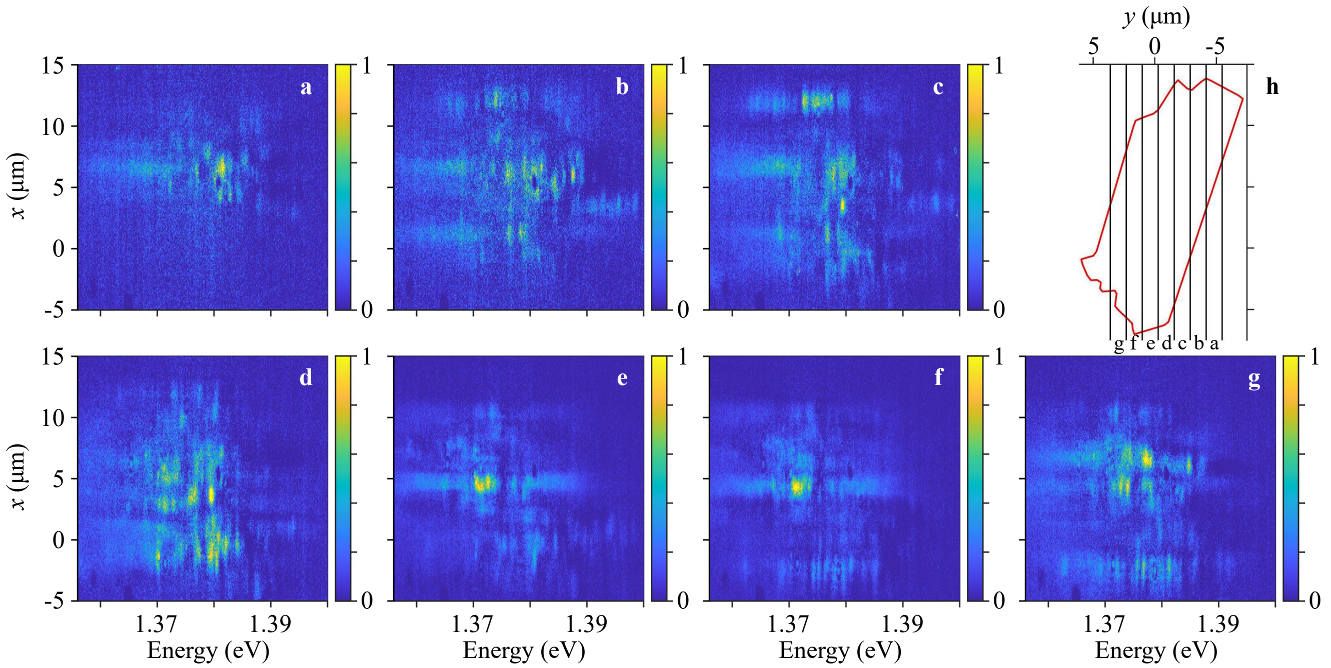

FIG. S7: (a-g) x−Energy maps of narrow lines for $y = -4.8$ μm (a), $y = -3.5$ μm (b), $y = -2.2$ μm (c), $y = -0.9$ μm (d), $y = 0.4$ μm (e), $y = 1.7$ μm (f), and $y = 3$ μm (g). The signal is integrated within 1.3 μm in y direction. The broad background (given by Gaussians in Fig. 1a in the main text) is subtracted. x−Energy maps without background subtraction are shown in Fig. S8. The excitation spot is defocused over a spot ~ 25 μm in diameter covering the heterostructure area for a weak excitation of the entire sample. The excitation power of this defocused excitation is 50 μW. $T = 4.2$ K. (h) $x - y$ map of the sample showing the y positions of the slit and the 1.3 μm ranges of the signal integration in the y direction given by the slit for the x−Energy maps in (a-g). The boundary of the $MoSe_2/WSe_2$ heterostructure is shown by the red line.

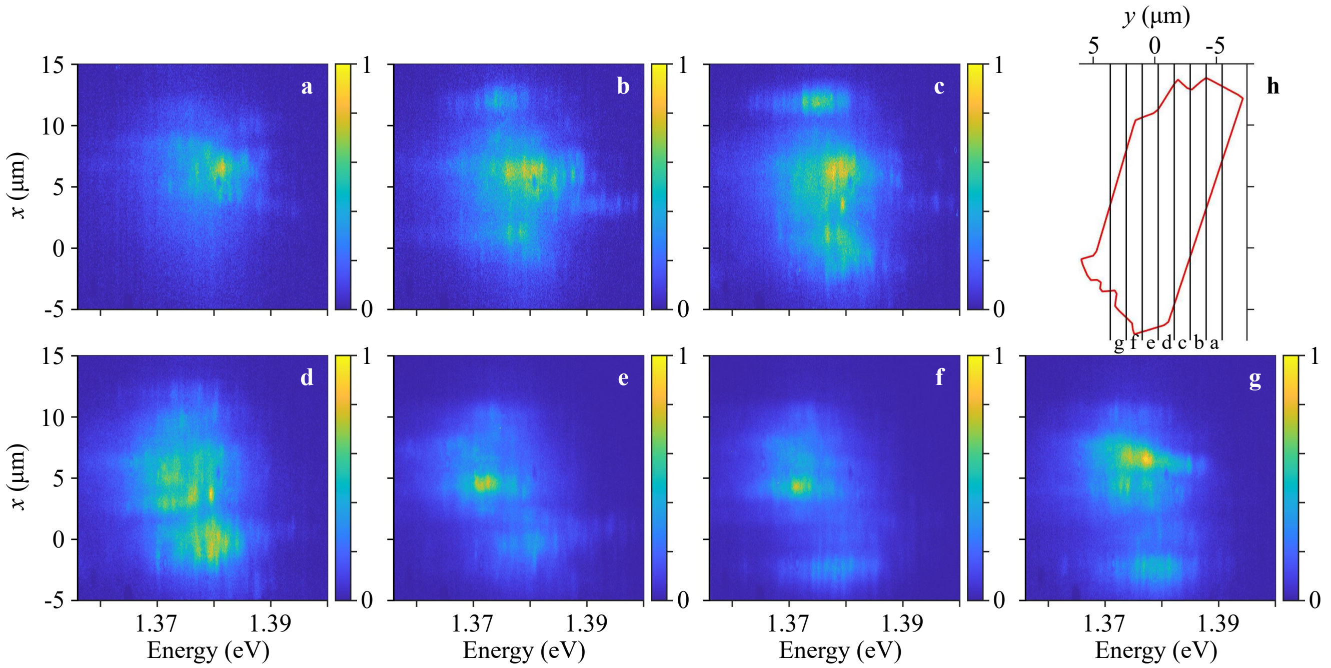

FIG. S8: x−Energy maps of narrow lines similar to the maps in Fig. S7, however, with no subtraction of the broad background.

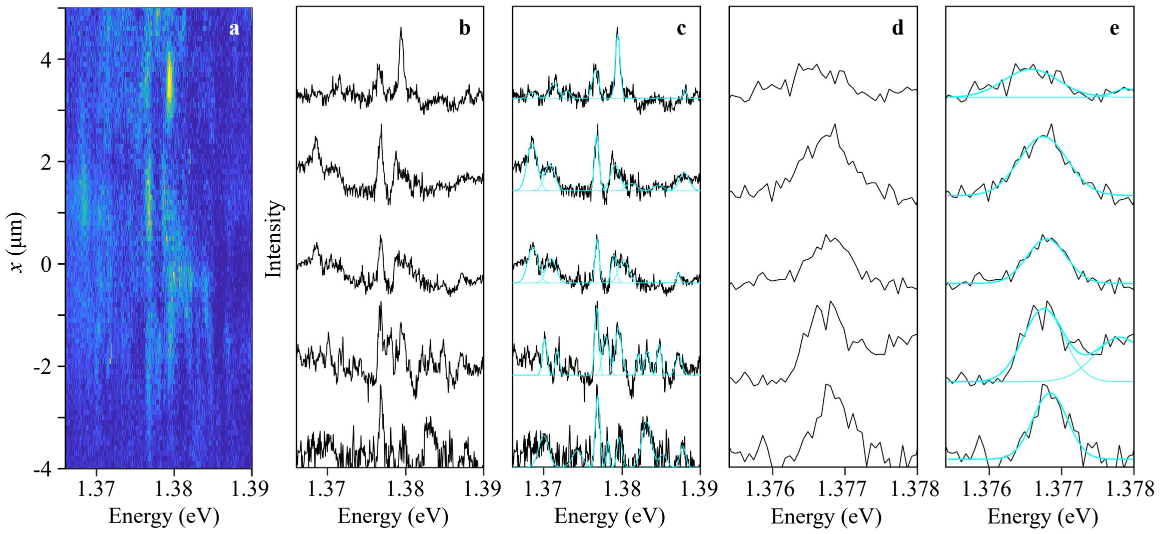

FIG. S9: (a) x−Energy map of narrow lines for $y = -2.2$ μm (from Fig. S7c). (b) The spectra of narrow lines corresponding to this x−Energy map at $x = 4$ μm, $x = 1.5$ μm, $x = 0.7$ μm, $x = -2.3$ μm, and $x = -3.5$ μm (from top to bottom). The highest intensities are normalized. (c) Same as (b) with the cyan lines showing the Gaussian fits to the narrow lines. (d) The parts of the spectra in (b) showing the lineshape of the narrow line at 1.3768 eV for various x positions. (e) Same as (d) with the cyan lines showing the Gaussian fits to the narrow lines.

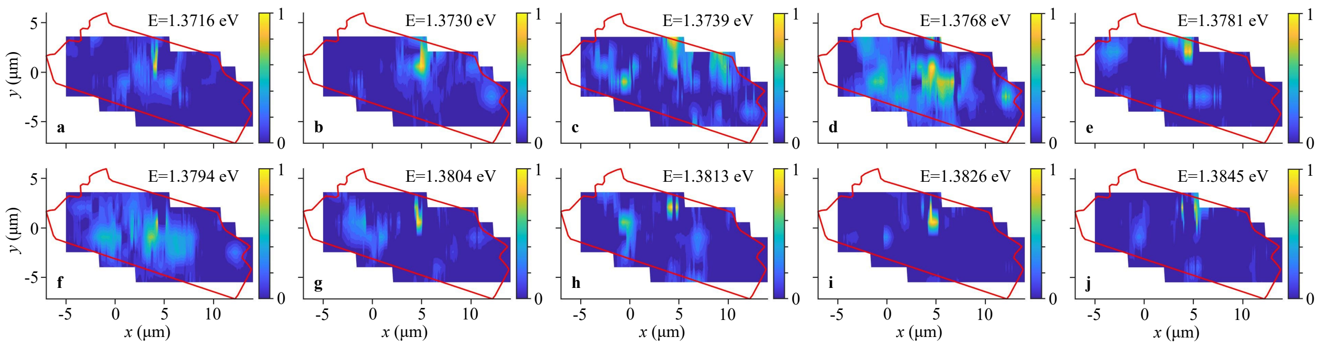

FIG. S10: $x - y$ maps showing the spatial extension of the exciton states corresponding to the narrow lines. The energies of the narrow lines are indicated. The signal is collected above the broad background (given by Gaussians in Fig. 1a in the main text) within the 1 meV linewidth of the narrow line. The boundary of the $MoSe_2/WSe_2$ heterostructure is shown by the red line. The excitation spot is defocused over a spot ~ 25 μm in diameter covering the heterostructure area for a weak excitation of the entire sample. The excitation power of this defocused excitation is 50 μW. $T = 4.2$ K.

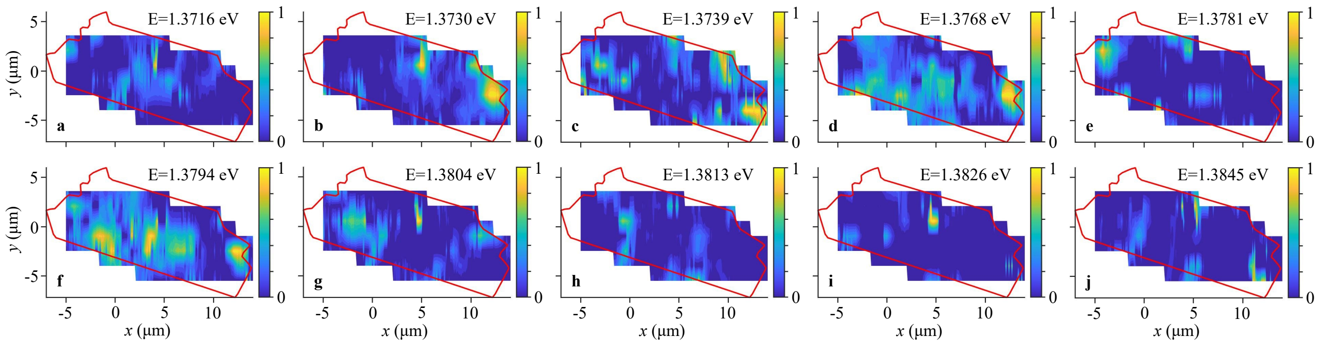

FIG. S11: $x - y$ maps of narrow lines similar to the maps in Fig. S10, however, with the intensity of the narrow line normalized by the intensity of the broad-line background at each location.

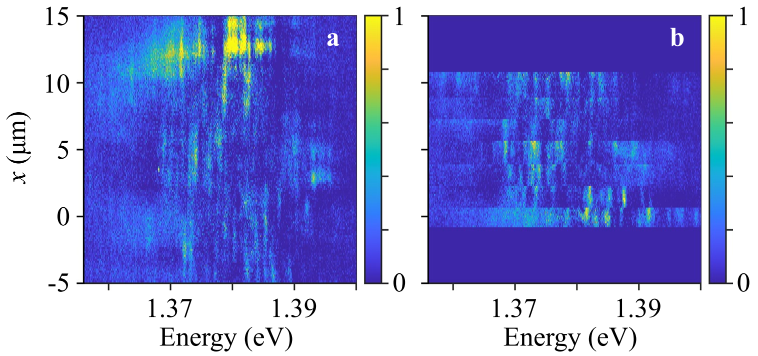

FIG. S12: x−Energy map of narrow lines. (a) The defocused laser excitation allows obtaining x−Energy map of narrow lines without moving the laser excitation spot. (b) Using the focused laser excitation and measuring the spectra at various x positions of the laser excitation spot allows reconstructing x−Energy map of narrow lines, however the accuracy is lower. The broad background is subtracted. For the defocused laser excitation in (a), the excitation spot size is $\sim 35 \times 45$ μm, $P_{ex} = 200$ μW. For the focused laser excitation in (b), the excitation spot size is ~ 2 μm in diameter, $P_{ex} = 10$ μW. The focused laser excitation spot is moved to the positions $x = 0$ μm, $x = 1.5$ μm, $x = 3.1$ μm, $x = 4.7$ μm, $x = 6.8$ μm, $x = 7.8$ μm, and $x = 10$ μm and the spectra measured at these x are shown at the corresponding positions on the x−Energy map in (b). $T = 4.2$ K.

$x - y$ maps

Figure S10 shows the $x - y$ maps for the exciton states corresponding to the narrow lines. This figure is similar to Fig. 3 in the main text, however, it shows $x - y$ maps for more narrow lines in the heterostructure.

Figure S11 shows $x - y$ maps of narrow lines similar to the maps in Fig. S10. For the data in Fig. S11, the broad background is subtracted as for the data in Fig. S10. However, while Fig. S10 shows the $x - y$ map of intensity of the narrow line, Fig. S11 shows the $x - y$ map of intensity of the narrow line normalized by the intensity of the broad background at each location. The normalization corrects for signal variations, e.g. due to absorption on the surface, which may contain imperfections.

Focused excitation

For the x-Energy maps in Fig. 2 in the main text and in Figs. S3, S4, S7-S9, we used a defocused laser excitation covering the entire heterostructure. The defocused laser excitation allows obtaining x-Energy maps of narrow lines without moving the laser excitation spot (Fig. S12a). For the data in Fig. S12b, we used a laser beam focused to a spot ~ 2 μm in diameter and moved it in the x direction to obtain spectra for various x positions. Similar to the spectra obtained with the defocused laser excitation, the spectra obtained with the focused laser excitation show narrow lines, which are revealed by the spectrally narrow enhancements of the PL intensity (Fig. S12). The spectra obtained with the focused laser excitation at various x positions allow reconstructing an x−Energy map (Fig. S12b), however, the accuracy of this map is lower than the accuracy of the x−Energy map measured with the defocused laser excitation where moving and precise positioning of the laser beam is not needed (Fig. S12a).

References

[1] F. Withers, O. Del Pozo-Zamudio, A. Mishchenko, A.P. Rooney, A. Gholinia, K.Watanabe, T. Taniguchi, S.J. Haigh, A.K. Geim, A.I. Tartakovskii, K.S. Novoselov, Light-emitting diodes by band-structure engineering in van der Waals heterostructures, *Nat. Mater.* **14**, 301 (2015).

[2] L.H. Fowler-Gerace, Zhiwen Zhou, E.A. Szwed, D.J. Choksy, L.V. Butov, Transport and localization of indirect excitons in a van der Waals heterostructure, *Nat. Photonics* **18**, 823 (2024).

[3] Zhiwen Zhou, E.A. Szwed, D.J.Choksy, L.H. Fowler-Gerace, L.V. Butov, Long-distance decay-less spin transport in indirect excitons in a van der Waals heterostructure, *Nat. Commun.* 15, 9454 (2024).

[4] Zhiwen Zhou, W.J. Brunner, E.A. Szwed, H. Henstridge, L.H. Fowler-Gerace, L.V. Butov, Efficient transport of indirect excitons in a van der Waals heterostructure, arXiv:2507.04556 (2025).

[5] Kyle L. Seyler, Pasqual Rivera, Hongyi Yu, Nathan P. Wilson, Essance L. Ray, David G. Mandrus, Jiaqiang Yan, Wang Yao, Xiaodong Xu, Signatures of moiré-trapped valley excitons in $MoSe_2/WSe_2$ heterobilayers, *Nature* **567**, 66 (2019).

[6] Tomasz Woźniak, Paulo E. Faria Junior, Gotthard Seifert, Andrey Chaves, Jens Kunstmann, Exciton g factors of van der Waals heterostructures from first-principles calculations, *Phys. Rev. B* **101**, 235408 (2020).
